# A Randomized Controlled Trial Comparing the Quality of Life and Medication Adherence in Patients on Antidepressant Monotherapy

**DOI:** 10.7759/cureus.62418

**Published:** 2024-06-15

**Authors:** N Simple Santi, Sashi B Biswal, Birendra Narayan Naik, Jyoti Prakash Sahoo, Bhabagrahi Rath

**Affiliations:** 1 Pharmacology, Veer Surendra Sai Institute of Medical Sciences and Research (VIMSAR), Burla, IND; 2 Psychiatry, Veer Surendra Sai Institute of Medical Sciences and Research (VIMSAR), Burla, IND; 3 Pharmacology, Kalinga Institute of Medical Sciences, Bhubaneswar, IND

**Keywords:** short form-36, medication adherence, quality of life, selective serotonin reuptake inhibitor (ssri), escitalopram, vilazodone, vortioxetine, morisky medication adherence scale-8 (mmas-8), hamilton depression rating scale, depressive disorders

## Abstract

Background and objectives

The quality of life declines with the growing severity of major depressive disorder (MDD). In depressed people, medication adherence and the quality of life are mutually corrosive. These concerns spurred the investigation of relationships between treatment outcomes and adherence levels. Limited studies are looking at how vortioxetine, escitalopram, and vilazodone affect these parameters. We aimed to detect how the Short Form-36 (SF-36) had changed 16 weeks after the baseline. The connection between treatment results (as expressed by the Hamilton Depression Rating Scale or HDRS) and medication adherence (as reflected by the Morisky Medication Adherence Scale-8 or MMAS-8) was also explored.

Methods

An open-label, randomized, three-arm trial with 96 MDD patients was conducted. For 16 weeks, the participants were put into three groups per a 1:1:1 ratio and administered tablets of vilazodone (20-40 mg/day), escitalopram (10-20 mg/day), or vortioxetine (5-20 mg/day). There were two test drugs: vilazodone and vortioxetine; the control was escitalopram. Four weeks apart, follow-up appointments were set after the baseline visit. The HDRS, mental and physical components of SF-36, and MMAS-8 scores were evaluated in the per-protocol (PP) population. Reduced HDRS scores were indicative of improved depression symptoms. Higher MMAS-8 and SF-36 scores indicated high drug adherence and enhanced quality of life. Our analysis used the Kruskal-Wallis test, the Bonferroni correction, and the Sankey diagram. In the Clinical Trial Registry-India (CTRI), we recorded this study prospectively (2022/07/043808).

Results

One hundred nine (81.34%) of the 134 individuals we examined were eligible. The PP population consisted of 96 (88.07%) of them who wrapped up the 16-week study. The mean age of the group was 46.3 ± 6.2 years. For each of the three groups, the SF-36 physical component scores revealed a median difference of 24.5 (23.8-26.0), 24.0 (22.8-25.3), and 27.0 (25.0-29.0) (p = 0.001). Accordingly, the mental components of their SF-36 scores showed a median difference of 32.0 (31.0-33.3), 31.0 (29.8-34.3), and 36.0 (33.0-38.0) (p = 0.001). A median difference of -15.0 (-16.0 to -14.0), -16.0 (-17.0 to -15.0), and -16.0 (-17.0 to -15.8) was observed in the HDRS scores after 16 weeks, with respect to the baseline (p < 0.001). The median MMAS-8 scores at 16 weeks were 6.0 (6.0-7.0), 6.8 (6.0-7.0), and 7.5 (6.5-8.0) (p = 0.031). The Sankey diagram illustrated the connection between better treatment results, increased medication compliance, and decreased symptoms of depression.

Conclusion

In comparison to vilazodone and escitalopram, vortioxetine demonstrated a statistically significant decrease in HDRS scores and an improvement in the physical and mental component scores of the SF-36. Clinical improvements were evident in the individuals' drug adherence levels. Larger-scale studies are advised to investigate the effects of these medications on the quality of life, medication adherence, and treatment outcomes.

## Introduction

One of the most incapacitating conditions in the world, major depressive disorder (MDD), has negative influences on the quality of life, cognitive function, and psychological health in general [1]. Worldwide, 322 million people are reckoned to be depressed [2,3]. There is an increasing prevalence of MDD in younger people [1]. From 1990 to 2017, there was a 50% surge in the global incidence of depressive illnesses [4]. Of Indians, 15.9% struggle with depressive illnesses each year, as per studies published lately [5,6].

The repercussions of MDD on mental, social, and metabolic health are deleterious [6-8]. Slowly but surely, the depressed patient's compliance and quality of life deteriorate as their condition deepens. At this point, MDD management is considered to hinge on adherence to therapy with antidepressants [9-11]. The five pillars of health-related quality of life (HRQoL) are perceptions of one's health, total life satisfaction and well-being, and psychological, physical, and social wellness [12].

We picked three antidepressants: vortioxetine, a regulator of serotonin receptors and transporters; escitalopram, a selective serotonin receptor inhibitor (SSRI); and vilazodone, an SSRI plus 5-HT_1A_ partial agonist. A high rate of treatment compliance yields effective mitigation of depressive symptoms along with enhanced HRQoL, according to some recent studies [13-15]. The study at hand was spurred by the assumption that antidepressants with unique mechanisms of action would render a convincing option for those diagnosed with MDD. The study's interim analysis substantiated these pharmaceuticals' safety, efficacy, and effect on the quality of life [3,6,8,16]. Beneficial outcomes were reported concerning these medications' safety and efficacy when used alone [17].

Following 16 weeks of antidepressant monotherapy with the drugs mentioned above, we mapped to gauge medication adherence and the quality of life in MDD patients. Here, we present changes in the physical and mental components of Short Form-36 (SF-36) [18]. At 16 weeks, we additionally explored the relationship between adherence (quantified with the Morisky Medication Adherence Scale-8 {MMAS-8}) [19] and the variations in the Hamilton Depression Rating Scale (HDRS) [20].

## Materials and methods

This was an active-controlled, randomized, three-arm, open-label study. For patients with MDD receiving monotherapy with vilazodone, vortioxetine, and escitalopram, we measured their quality of life and adherence to their treatment regimens. From July 2022 to December 2023, the research was carried out at the Veer Surendra Sai Institute of Medical Sciences and Research (VIMSAR) in Burla, India. The Institutional Research and Ethics Committee granted us ethical approval (029-2022/I-S-T/03) before we started the trial. Before the enrollment process, all participants gave their written informed consent. A prospective registration for our study was made in the Clinical Trial Registry-India (CTRI) (2022/07/043808). The study complied with the Declaration of Helsinki, institutional policies, and good clinical practices.

Study participants

The participants diagnosed with MDD, together with an HDRS score of 24 or more, were included in our study. Any disclosed allergy to study medications, chronic kidney failure, organic brain disorders, symptoms of psychosis, abnormal lipid profiles, and thromboembolic events within the previous six months were all considered exclusion criteria, nor did this study include mothers who were nursing or pregnant. The participants could always withdraw their consent at any time.

Study design and endpoints

The experimental drugs in this trial were vilazodone and vortioxetine, while the control was escitalopram. To ensure randomization, those deemed eligible were divided into three groups: group A received vilazodone (20-40 mg), group B received escitalopram (10-20 mg), and group C received vortioxetine (5-20 mg). The allotment ratio was 1:1:1. We used permuted block randomization using blocks of 12 and 24 sizes. Based on the gender and duration of MDD, we stratified the randomization.

The primary objective was to determine the changes in the physical and mental aspects of SF-36 from the baseline at week 16. The secondary objectives comprised the MMAS-8 score at week 16 and the implication of medication adherence level on treatment outcome measured through HDRS. The per-protocol (PP) sample was the focus of our evaluations.

Study procedure

Throughout the trial, the individuals got monotherapy, that is, just their prescribed drugs. Each of them received a daily dosage of oral medications for 16 weeks. It was not allowed to switch between study medications and other antidepressants. Once the psychiatrist evaluated the patient's response to the medication, the recommended regimen was modified. During the baseline visit, each participant was carefully assessed psychologically and physically. Follow-up appointments were scheduled for the subjects at four, eight, 12, and 16 weeks following the start of therapy.

The quality of life of each participant was assessed for modifications using SF-36 [18]. The eight sections of this questionnaire are designed to evaluate both the physical and mental aspects of an individual's quality of life. These include vitality, bodily functioning, discomfort, mental well-being, perceptions of general health, and social, emotional, and physical role functioning. The SF-36 scale produces eight scaled scores, which are derived from the weighted sums of the answers to the questions in each section. From zero to 100 is the range of scores. Greater quality of life and less disability are represented by higher scores.

Implementing the MMAS-8 [19], their medication adherence was assessed. The total score falls between zero and eight. The numbers <6, 6-7, and 8 indicate low, moderate, and high medication adherence in that order. Furthermore, we probed the interaction between the level of adherence at 16 weeks and the clinical outcomes gauged by the difference in HDRS [20] scores from baseline at week 16. Depressive symptoms are lessened, and treatment outcomes tend to improve when HDRS scores are lowered.

Statistical analysis

We assumed a mean difference of 10.0 in HDRS from baseline values coupled with a standard deviation (SD) of 2.0 to determine the sample size. A two-sided alpha error of 0.05 and a beta error of 0.20 (i.e., 80% power) were obligated for 87 cases. Ninety-six cases were the sample size that we chose to allow for a 10% dropout rate. An interim analysis was carried out following the completion of the 12-week visits for the first 48 participants.

We confirmed the normality of the data distribution using the Shapiro-Wilk test. The summary statistics for the qualitative data were proportion and frequency. The mean and standard deviation (SD), or median and interquartile range (IQR), were adopted to portray the quantitative data. Using Pearson's chi-square test, we evaluated the sociodemographic features. The Kruskal-Wallis test gauged the HDRS, SF-36, and MMAS-8 scores. For post hoc analysis, the Bonferroni test was selected. We relied on R (4.3.3) (R Foundation for Statistical Computing, Vienna, Austria) [21] for data analysis and visualization. Two-tailed statistical tests were used. For p-values of less than 0.05, statistical significance was explained.

## Results

**Figure 1:**
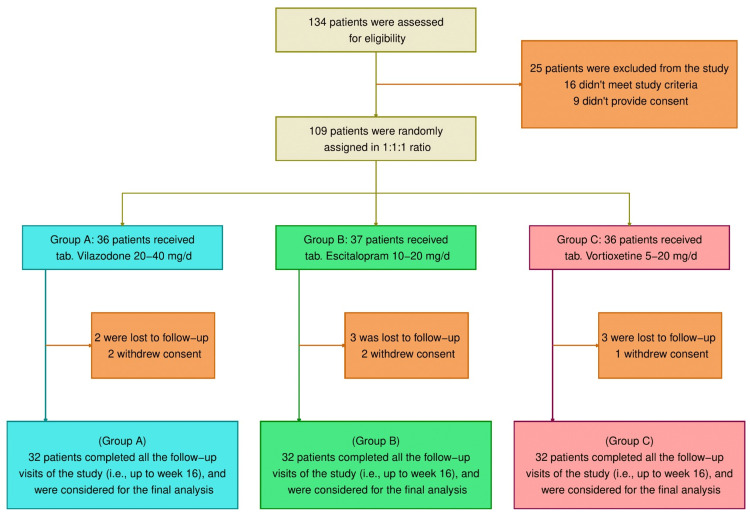
CONSORT diagram Group A, vilazodone; Group B, escitalopram; and Group C, vortioxetine CONSORT: Consolidated Standards of Reporting Trials

A total of 134 patients had their eligibility assessed (Figure 1). Of them, 16 were ineligible, and nine denied their participation. Those 25 participants were dropped out of the study. The remaining 109 patients were placed arbitrarily in one of the three study groups. Eight did not follow up, while five revoked their consent. The 96 participants who finished all follow-up appointments until week 16 underwent evaluations. Similar baseline demographic features were evident in all three groups (Table 1).

**Table 1:** Baseline sociodemographic traits of the study population (n = 96) The median (IQR) or the mean ± SD was chosen to depict the continuous variables. N (%) was used to display the category values. T/t naïve: a newly diagnosed or treatment-naïve patient BMI, body mass index; HDRS, Hamilton Depression Rating Scale (17-item version); SF-36, Short Form-36; SD, standard deviation; IQR, interquartile range

Parameters	Total (n = 96)	Group A: vilazodone (n = 32)	Group B: escitalopram (n = 32)	Group C: vortioxetine (n = 32)	P-value
Age (years)	46.3 ± 6.2	47.1 ± 6.4	46.0 ± 5.5	45.7 ± 6.1	0.143
Age group					
≤50 years	64 (66.7%)	23 (71.9%)	20 (62.5%)	21 (65.6%)	0.580
>50 years	32 (33.3%)	9 (28.1%)	12 (37.5%)	11 (34.4%)	
Gender					
Female	48 (50.0%)	16 (50.0%)	16 (50.0%)	16 (50.0%)	1
Male	48 (50.0%)	16 (50.0%)	16 (50.0%)	16 (50.0%)	
Literacy					
Literate	80 (83.3%)	27 (84.4%)	26 (81.2%)	27 (84.4%)	0.189
Illiterate	16 (16.7%)	5 (15.5%)	6 (18.8%)	5 (15.5%)	
Marital status					
Married	72 (75.0%)	25 (78.1%)	23 (71.9%)	24 (75.0%)	0.477
Unmarried	24 (25.0%)	7 (21.9%)	9 (28.1%)	8 (25.0%)	
Duration of disease					
T/t naïve	48 (50.0%)	16 (50.0%)	16 (50.0%)	16 (50.0%)	1
<6 months	48 (50.0%)	16 (50.0%)	16 (50.0%)	16 (50.0%)	
BMI (kg/m^2^)	27.3 ± 4.8	26.4 ± 4.1	27.7 ± 5.2	27.8 ± 4.5	0.028
HDRS	30.0 (29.0-31.0)	30.0 (29.0-31.0)	30.0 (29.0-31.0)	30.0 (29.0-31.2)	0.964
SF-36 (physical component)	35.0 (34.0-36.0)	35.0 (34.0-36.0)	35.0 (34.8-36.0)	35.0 (34.0-36.0)	0.547
SF-36 (mental component)	43.0 (42.0-44.0)	43.0 (42.0-44.0)	43.5 (42.0-45.0)	43.0 (42.0-44.0)	0.367

**Figure 2:**
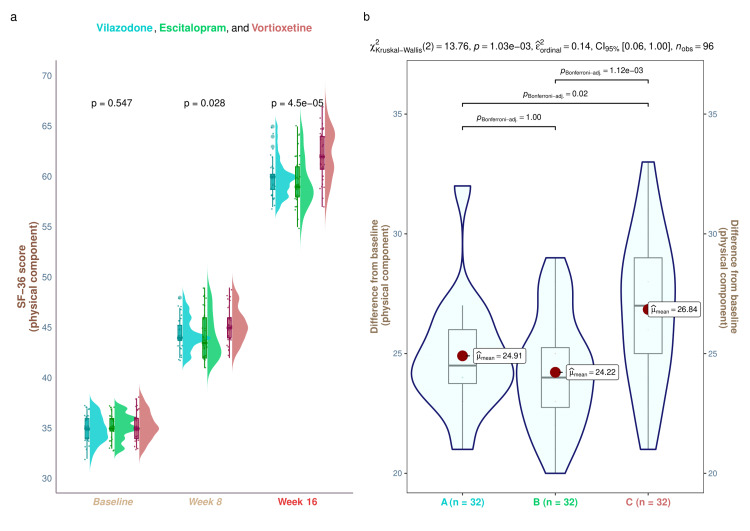
The physical component scores of SF-36 of the study participants These graphs show the SF-36 physical component scores for the three groups' participants. (a) The SF-36 physical component scores at baseline and weeks 8 and 16 are displayed as the raincloud plots. Intergroup analyses were done by running the Kruskal-Wallis test. (b) The post hoc assessment of the variations from the baseline is shown. It was evaluated via the Bonferroni method SF-36: Short Form-36

**Figure 3:**
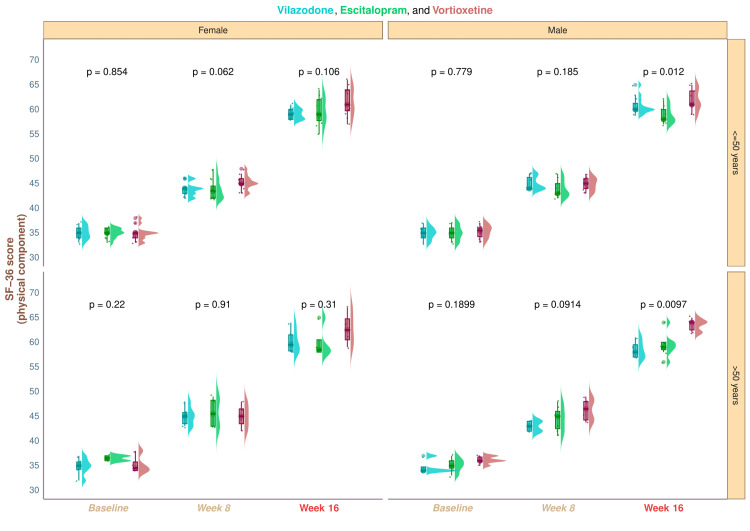
Subgroup analysis of the physical component scores of SF-36 The subgroup analysis of the participants' SF-36 physical component scores across all three groups is depicted in this figure. The vertical and horizontal grids, respectively, display the age (≤50 and >50 years) and gender (female and male). We contrasted the data using the Kruskal-Wallis test SF-36: Short Form-36

The three study groups' median baseline scores of the physical component of SF-36 were 35.0 (34.0-36.0), 35.0 (34.8-36.0), and 35.0 (34.0-36.0), respectively (p = 0.547). The equivalent scores were 44.0 (43.8-45.2), 43.5 (42.0-46.0), and 45.0 (44.0-46.0) after eight weeks of treatment (p = 0.028). After the 16-week therapy, the medians increased to 58.8 (60.0-60.2), 59.0 (58.0-61.0), and 62.0 (60.8-64.0), respectively (p < 0.001). We observed a statistically significant increase (p < 0.001) in the quality of life in each of the study groups, as indicated by the physical component of SF-36 (Figure 2a). These data show that following a 16-week intervention, the research population's overall quality of life rose, and the frequency and severity of depressive symptoms declined. There were statistically significant differences (p = 0.001) between the groups when comparing the changes from baseline scores. We conducted the post hoc analysis using the Bonferroni correction. The results showed a statistically significant difference between the vilazodone (p = 0.02) and escitalopram (p = 0.001) groups of patients and the vortioxetine group (Figure 2b). Regardless of age, male participants in the vortioxetine group demonstrated statistically significant improvements over those in the other two groups in their quality of life assessments (younger male, p = 0.012; elderly male, p = 0.0097). Nevertheless, there was no statistically significant difference between the groups of female participants (older female, p = 0.31; younger female, p = 0.106) (Figure 3).

**Figure 4:**
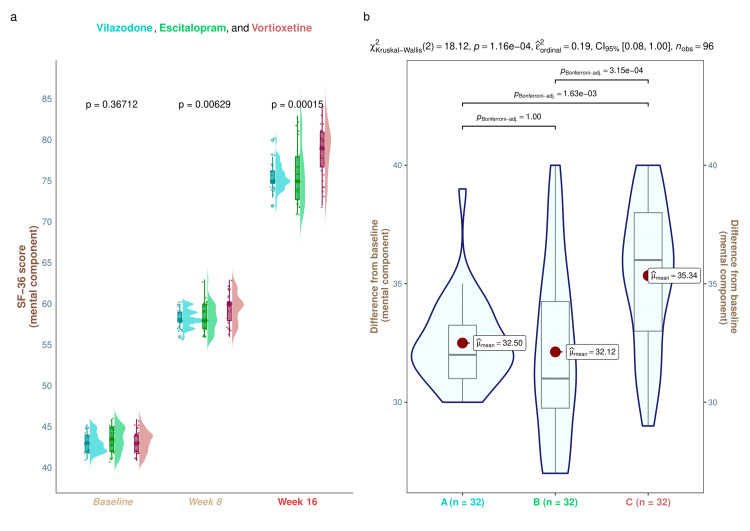
The mental component scores of SF-36 of the study participants These graphs show the SF-36 mental component scores for the three groups' participants. (a) the SF-36 mental component scores at baseline and weeks 8 and 16 are displayed as the raincloud plots. Intergroup analyses were done by running the Kruskal-Wallis test. (b) The post hoc assessment of the variations from the baseline is shown. It was evaluated via the Bonferroni method SF-36: Short Form-36

**Figure 5:**
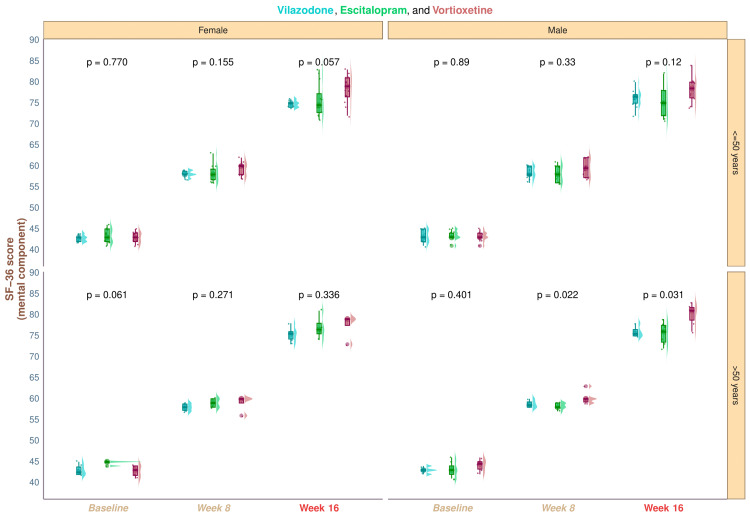
Subgroup analysis of the mental component scores of SF-36 The subgroup analysis of the participants' SF-36 mental component scores across all three groups is depicted in this figure. The vertical and horizontal grids, respectively, display the age (≤50 and >50 years) and gender (female and male). We contrasted the data using the Kruskal-Wallis test SF-36: Short Form-36

The three study groups' median baseline scores of the mental component of SF-36 were 43.0 (42.0-44.0), 43.5 (42.0-45.0), and 43.0 (42.0-44.0) (p = 0.367). The corresponding scores were 58.0 (58.0-59.0), 58.0 (57.0-60.0), and 60.0 (58.0-60.2) after eight weeks of treatment (p = 0.006). The medians changed after a 16-week period to 75.0 (74.8-76.2), 75.0 (72.8-78.0), and 79.0 (76.8-81.0) (p < 0.001). In each of our study groups, we detected a statistically significant improvement in the quality of life, as suggested by the mental component scores of SF-36 (p < 0.001) (Figure 4a). These numbers imply that following a 16-week intervention, the study population had a decrease in the severity of symptoms of depression, as well as improved quality of life. Statistically significant differences (p < 0.001) were seen in intergroup comparisons regarding the changes from baseline scores. We conducted the post hoc analysis using the Bonferroni correction. It disclosed that there was a statistically significant difference (p < 0.001) between the group receiving vortioxetine and the groups receiving vilazodone and escitalopram (Figure 4b). Subgroup analysis revealed that elderly males in the vortioxetine group had statistically significant improvement in the quality-of-life scores (p = 0.031) compared to the other two groups' participants. However, the intergroup differences did not show statistical significance for the younger male (p = 0.12) and all female participants (elderly female, p = 0.336; younger female, p = 0.057) (Figure 5).

The antidepressant effects of the drugs were gauged with HDRS. The medication adherence was weighed using MMAS-8 (Table 2). All three drugs' HDRS scores plummeted after 16 weeks of intervention. The table shows that the intergroup differences in HDRS scores became statistically more significant with time. The differences at 16 weeks from baseline were clinically and statistically significant (p < 0.001). Escitalopram and vortioxetine's HDRS scores were reduced by >15 in the majority of the participants. Most participants (54) showed moderate medication adherence (MMAS-8 score = 6-7). Twenty-seven adhered highly (MMAS-8 score = 8) to the study medications. The three study groups' median MMAS-8 scores at week 16 were 6.0 (6.0-7.0), 6.8 (6.0-7.0), and 7.5 (6.5-8.0), respectively (p = 0.031).

**Table 2:** HDRS and MMAS-8 scores in the study population (n = 96) HDRS and MMAS-8 scales were leveraged to assess the treatment outcome and medication adherence. The p-values for the categorical and continuous data were calculated using the chi-square (χ^2^) and Kruskal-Wallis tests, respectively MMAS-8, Morisky Medication Adherence Scale-8; HDRS, Hamilton Depression Rating Scale

Parameters	Group A: vilazodone (n = 32)	Group B: escitalopram (n = 32)	Group C: vortioxetine (n = 32)	P-value
Treatment outcome (assessed with HDRS)				
HDRS score at baseline	30.0 (29.0-31.0)	30.0 (29.0-31.0)	30.0 (29.0-31.2)	0.964
HDRS score at four weeks	27.0 (26.0-28.3)	27.0 (26.0-28.0)	26.0 (25.0-28.2)	0.581
HDRS score at eight weeks	24.0 (23.0-25.0)	23.5 (23.0-24.0)	23.0 (22.0-24.0)	0.064
HDRS score at 12 weeks	20.0 (18.0-21.0)	20.0 (19.0-20.2)	19.0 (18.0-20.0)	0.058
HDRS score at 16 weeks	15.0 (14.0-16.0)	14.0 (13.0-15.0)	13.0 (13.0-15.0)	0.002
Difference in HDRS from baseline	-15.0 (-16.0 to -14.0)	-17.0 (-16.0 to -15.0)	-17.0 (-16.0 to -15.8)	<0.001
Difference in HDRS of >15 from baseline, n (%)	9 (28.1%)	21 (65.6%)	24 (75.0%)	0.001
Medication adherence (assessed with MMAS-8)				
MMAS-8 score at 16 weeks	6.0 (6.0-7.0)	6.8 (6.0-7.0)	7.5 (6.5-8.0)	0.031
High adherence (score = 8), n (%)	4 (12.5%)	9 (28.1%)	14 (43.8%)	0.025
Moderate adherence (score = 6-7), n (%)	21 (65.6%)	20 (62.5%)	13 (40.6%)	0.187
Low adherence (score < 6), n (%)	7 (21.9%)	3 (9.4%)	5 (15.6%)	0.329

**Figure 6:**
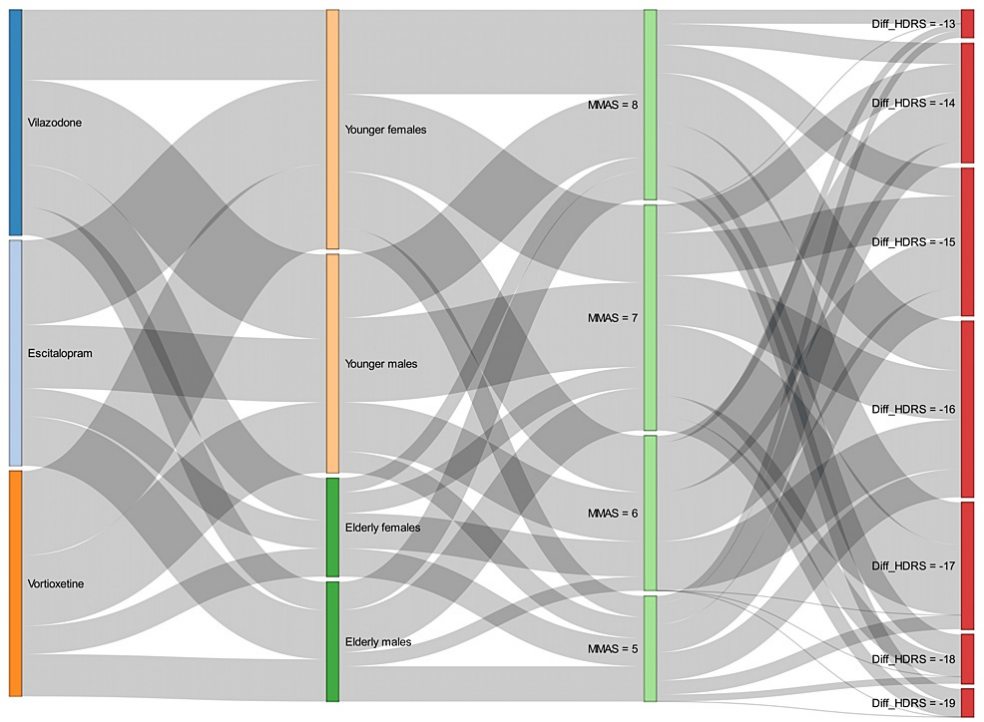
The Sankey diagram illustrating the association of medication adherence and differences in HDRS scores in the study population The study groups are shown in the first column. The participants were categorized as per their gender and age group (younger, ≤50 years; elderly, >50 years) in the second column. The MMAS-8 scores at week 16 are illustrated in the third column, which denotes the medication adherence levels. The changes in HDRS scores from baseline at week 16 are depicted in the fourth column. The width of the connecting band between any two parameters indicates their degree of association MMAS-8, Morisky Medication Adherence Scale-8; HDRS, Hamilton Depression Rating Scale (17-item version)

The Sankey diagram in Figure 6 portrays the association between the medication adherence levels and the difference in HDRS scores at 16 weeks from baseline. The four columns represent the three study groups, study participants (based on age group and gender), MMAS-8 scores at week 16, and difference in HDRS score at week 16 from baseline, respectively. The width of the connecting bands is proportional to the number of participants with the concerned parameters. The younger participants were more numerous than the older people. The plot depicted that most participants were moderately adherent to the treatment, as suggested by the MMAS-8 scores (i.e., 6-7) at week 16. The MMAS-8 scores at the final visit spanned from 5 to 8. The differences in HDRS scores from the baseline ranged from -19 to -13. The participants with moderate to high adherence levels had a pronounced reduction in HDRS scores at week 16. Therefore, better treatment outcomes could be anticipated among the participants with high medication adherence.

## Discussion

Though all three drugs showed clinical improvement in physical and mental component scores of SF-36 at 16 weeks, the differences between vortioxetine and the other two medications were statistically significant. After 16 weeks of intervention, the differences in HDRS scores were consistent with medication adherence. This study's interim analysis [6] also displayed similar findings.

The two test drugs chosen for this trial were vilazodone (20-40 mg daily) and vortioxetine (5-20 mg daily). Escitalopram (10-20 mg) was dosed daily in the control group. Vilazodone's 5-HT_1A_ receptor partial agonistic activity may be the reason for its added benefit over escitalopram. Conversely, vortioxetine inhibits the transporters and blocks serotonin receptors. According to our previous paper [17], which compared the safety and effectiveness of these three medications, vortioxetine demonstrated statistically significant decreases in HDRS and MADRS scores. In this instance as well, vortioxetine significantly improved the quality of life. In light of these results, vortioxetine alone may be a beneficial treatment for MDD.

An article published by Florea et al. [22] suggests that vortioxetine-treated patients with MDD may show significant improvements in both the physical and mental domains of SF-36. Better quality of life, high compliance with medications, and frequent follow-up visits constitute prerequisites for effective antidepressant activity, according to our study's interim analysis [3,6]. Our results regarding the SF-36 and MMAS-8 scores agreed with those of two earlier studies [14,15]. During the trial, all of our study participants got free medications. It is plausible that the low attrition rate was responsible for the substantial rise in the SF-36 scores and the gradual improvement in depression-related symptoms over time. The quality of life was maximally enhanced for the elderly male participants in the vortioxetine group. The Sankey diagram clarified the association between medication adherence and a reduction in HDRS scores.

The primary positive aspects of this research were the evaluation of medication adherence using MMAS-8 [19], the quality of life using SF-36 [18], and a randomized study design with several follow-up visits. A couple of other aspects of our study could have been refined. First, the open-label design may have an impact on the outcomes. Second, antidepressants used in the trial were provided at no extra cost. The study results can be generalized if these drugs are affordably priced. Third, we could not perform the cost-effectiveness analysis. Fourth, there are numerous etiologic causes for depression, and it affects various aspects of the quality of life. In real-world settings, it might be tricky to verify each of these points in a patient on long-term antidepressant therapy.

## Conclusions

Vortioxetine significantly improved both the mental and physical components of SF-36 when weighed against escitalopram and vilazodone. Additionally, contrasted with the other two groups, there was a noticeable decline in the HDRS values with vortioxetine. Drug adherence statistics reflected the patients' clinical progress. We warrant further research with a larger sample size to evaluate these medications' long-term safety and efficacy. To ensure generalizability, more research must be conducted regarding their cost-effectiveness, effects on the quality of life, and medication adherence.
